# Antibacterial activity evaluation of selected essential oils in liquid and vapor phase on respiratory tract pathogens

**DOI:** 10.1186/s12906-018-2291-9

**Published:** 2018-07-27

**Authors:** Kamilla Ács, Viktória L. Balázs, Béla Kocsis, Tímea Bencsik, Andrea Böszörményi, Györgyi Horváth

**Affiliations:** 1 Department of Pharmacognosy, Faculty of Pharmacy, University of Pécs, Pécs, H-7624 Hungary; 2 Department of Medical Microbiology and Immunology, Medical School, University of Pécs, Pécs, Hungary; 3 Department of Pharmacognosy, Faculty of Pharmacy, Semmelweis University, Budapest, Hungary

**Keywords:** Vapor phase, Essential oil, Respiratory tract, Antibacterial activity, *Haemophilus* spp., *Streptococcus* spp.

## Abstract

**Background:**

The increasing number of multidrug-resistant bacteria and the fact of antibiotic resistance is leading to a continuous need for discovering alternative treatments against infections, e.g. in the case of respiratory tract diseases. Essential oils (EOs), because of their volatility, can easily reach both the upper and lower parts of the respiratory tract via inhalation. Therefore, the aim of the present study was the antibacterial evaluation of clove, cinnamon bark, eucalyptus, thyme, scots pine, peppermint, and citronella EOs against respiratory tract pathogens such as *Streptococcus pneumoniae*, *S. mutans*, *S. pyogenes*, *Haemophilus influenzae*, *H. parainfluenzae*, and *Moraxella catarrhalis*. Furthermore, we wanted to compare the antibacterial effect of these EOs in two different test systems to provide data for the development of an appropriate product formulation.

**Methods:**

Minimum inhibitory concentration (MIC) and minimum bactericidal concentration (MBC) were determined with in vitro vapor phase test (VPT) and broth macrodilution test (BDT). The chemical and percentage compositions of the EOs were determined by GC-MS and GC-FID analysis.

**Results:**

Among the EOs, thyme was the most effective against *S. mutans* (MIC: 0.04 mg/mL in BDT, but cinnamon bark and clove oils also presented high inhibition in liquid medium with MIC values of 0.06 mg/mL and 0.1 mg/mL against *S. pneumoniae* and *S. pyogenes*, respectively. *M. catarrhalis* was the most sensitive to thyme EO (MIC: 0.09 mg/mL). Cinnamon bark EO was the most effective against *Haemophilus* spp. (MIC: 0.06 mg/mL). In the VPT, cinnamon bark was the most effective oil against all investigated pathogens with MIC values in the range of 15.62–90 μl/L. Surprisingly, the eucalyptus and scots pine showed weak activity against the test bacteria in both test systems.

**Conclusions:**

The EO of thyme, clove and cinnamon bark may provide promising antibacterial activity against respiratory tract pathogens either in liquid medium or in vapor phase. However, their effect is lower than that of the reference antibiotics. The combination of EOs and antibiotics may be beneficial in the alternative treatment of respiratory tract diseases. In vivo studies are necessary to calculate the effective dose of EOs in patients and determine their possible side effects and toxicity.

## Background

Respiratory tract diseases cause significant mortality in both sexes according to the data of World Health Organization (WHO) [1]. Moreover, pneumonia was responsible for 13% of causes of death among post-neonatal (1–59 month) children in 2012 [2]. Several microorganisms are responsible for the upper/lower respiratory tract infections (RTIs). However, the number of studies including respiratory tract bacteria is low. Among antibiotic-resistant bacteria causing severe RTIs, this study focuses on the Gram-positive *Streptococcus mutans*, *S. pyogenes*, *S. pneumoniae*, and the Gram-negative *Haemophilus influenzae*, *H. parainfluenzae*, and *Moraxella catarrhalis*. The importance of the examination of these pathogenic bacteria is not questionable, because they frequently cause RTIs in humans.

RTIs include several acute or chronic diseases caused by viruses and/or bacteria. *H. influenzae* is responsible for e.g. epiglottitis. This bacterium together with *S. pneumoniae* and *M. catarrhalis* is able to evoke chronic bronchitis as well [3]. Among the lower RTIs, pneumonia is a highly dangerous infection, because it can easily lead to death. There are two major categories of pneumonias: community-acquired pneumonia and pneumonia associated with hospital, ventilation or health care. *H. influenzae* and *M. catarrhalis* are common in patients with community-acquired pneumonia, while *S. pneumoniae* occurs frequently among hospitalized patients [3].

There are a great number of products containing essential oils (EOs) in commercial marketing. However, EOs are generally applied based upon long-standing use in the complementary and alternative treatment of diverse diseases. Their antimicrobial potential is generally studied by several in vitro techniques, but mainly liquid phase is used in these assays instead of vapor phase (VP) [4]. The commonly used in vitro antimicrobial methods describe a wide range of assays with different parameters (agar recipes, incubation time, emulsifiers, microorganisms) [5, 6] so the results from the assays are very different and it is difficult to compare them. EOs are non-water-soluble substances, therefore, the commonly applied microbiological tests have been optimized to this condition.

In the case of RTIs, the vapor of EOs can pass into the airway and make a direct contact with the infected surface [7]. Therefore, it is worth investigating the antimicrobial effect of EOs in the VP. Previously, in vitro assessments of antimicrobial efficacy of EO vapors were published, however, there is no standardized in vitro VP method nowadays, and the comparison of the results of different studies is very difficult or even impossible [8–10]. The antimicrobial activity of EOs may alter among different in vitro conditions; therefore, the parallel assessment of this property in two test systems (liquid medium and VP) should provide more valuable results and data for development of new natural products used in the alternative treatment of RTIs.

Therefore, the aim of the present study was to evaluate the antibacterial effect of EOs of clove, cinnamon bark, eucalyptus, thyme, scots pine, peppermint, and citronella with in vitro broth macrodilution test (BDT) and vapor phase test (VPT) against pathogens responsible for both healthcare-associated and community-acquired RTIs. It should also be mentioned that bacteria included in this study have not been involved in VPT yet, except for *H. influenzae*.

## Methods

### Essential oil samples

The EOs of clove (*Syzygium aromaticum* (L.) Merill & Perry, Batch number: E0971/1211), cinnamon bark (*Cinnamomum zeylanicum* Nees., Batch number: A6302/0909), eucalyptus (*Eucalyptus globulus* Labill., Batch number: G1452/1404), thyme (*Thymus vulgaris* L., Batch number: E8392/1308), scots pine (*Pinus sylvestris* L., Batch number: G3032/1406), peppermint (*Mentha* × *piperita* L., Batch number: E7421/1307), and citronella (*Cymbopogon nardus* (L.) Rendle, Batch number: G3531/1407) were obtained from Aromax Ltd. (Budapest, Hungary).

### Headspace-solid phase microextraction (sHS-SPME) conditions

The chemical composition of the EOs was determined and published by our research group previously in 2016. The detailed conditions of GC-FID were described there [11]. Because the vapor of the EOs were used in the VPT, they were also analysed by sHS-SPME-GC-MS. sHS-SPME analysis is an effective and flexible analysis for the rapid characterization of the main components of the volatile fraction of plants [12]. In the method, 0.1 mL EO was put into a vial (20 mL headspace) sealed with a silicon/PTFE septum prior to SPME-GC-MS analysis. Using the static headspace solid phase microextraction technique, sample preparation was carried out with a CTC Combi PAL (CTC Analytics AG, Zwingen, Switzerland) automatic multipurpose sampler using a 65 μM StableFlex polydimethyl siloxane/divinyl benzene (PDMS/DVB) SPME fibre (Supelco, Bellefonte, PA, USA). After an incubation period of 5 min at 40 °C, extraction was performed by exposing the fibre to the headspace of a 20 mL vial containing the EO sample for 10 min at 40 °C. The fibre was then immediately transferred to the injector port of the GC-MS and desorbed for 1 min at 250 °C. The SPME fibre was cleaned and conditioned in a Fibre Bakeout Station in pure nitrogen atmosphere at 250 °C for 15 min.

### GC-MS conditions

The analyses were carried out with an Agilent 6890 N/5973 N GC-MSD (Santa Clara, CA, USA) system equipped with an Agilent SLB-5MS capillary column (30 m × 250 μm × 0.25 μm). The GC oven temperature was programmed to increase from 60 °C (3 min isothermal) to 250 °C at 8 °C/min (1 min isothermal). High purity helium was used as carrier gas at 1.0 mL/min (37 cm/s) in constant flow mode. The injector temperature was 250 °C. The split ratio was 1:50. The mass selective detector was equipped with a quadrupole mass analyser and it was operated in electron ionization mode at 70 eV in full scan mode (41–500 amu at 3.2 scan/s). The data were evaluated using MSD ChemStation D.02.00.275 software (Agilent). The identification of the compounds was carried out by comparing retention times, linear retention indexes and recorded spectra with the data of authentic standards, and the NIST 2.0 library was also consulted.

### Antimicrobial assays and chemicals

For the detection of antibacterial character of EOs, BDT and VPT were performed. The effect of EOs was also compared to the activity of standard antibiotics: imipenem (Fresenius Kabi, Hungary), amoxicillin/clavulanic acid (Richter Gedeon, Hungary), and amikacin (Lisapharma S.p.A., Italy). In VPT, we especially focused on the antibacterial effect of EO’ vapor. As solvent, absolute ethanol was obtained from Molar Chemicals Ltd. (Halásztelek, Hungary). The emulsifiers (Polysorbate 80, DMSO) were purchased from Reanal Ltd. (Budapest, Hungary). Mueller-Hinton agar, *Haemophilus* test medium base, and *Haemophilus* test supplement were purchased from Oxoid Ltd. (London, UK).

### Bacterial strains

The tests were performed against six bacterial strains including the most frequent respiratory tract pathogens. Gram-positive bacteria were *Streptococcus pneumoniae* (DSM 20566), *S. mutans* (DSM 20533), and *S. pyogenes* (116). Gram-negative strains included *Haemophilus influenzae* (DSM 4690), *H. parainfluenzae* (DSM 8978), and *Moraxella catarrhalis* (DSM 9143). *S. pyogenes* was isolated from blood cultures, it was used from the culture collection of the Department of Medical Microbiology and Immunology, Medical School, University of Pécs, Pécs, Hungary. All the other strains were obtained from the German Culture Collection (Braunschweig, Germany). Test microorganisms were maintained on 5% sheep blood agar or chocolate agar at 37 °C at the Department of Medical Microbiology and Immunology, University of Pécs (Pécs, Hungary). Antibiotic susceptibility of bacteria was tested by disc diffusion method according to the guidelines of the Manual of Clinical Microbiology [13]. To avoid the contamination of the test materials, EOs were filtered through a hydrophilic polyvinylidene fluoride (PVDF) membrane (Millex-GV filter, 0.22 μm, Millipore, Ireland) before the microbiological assays. The filtration did not modify the chemical composition of the EOs.

### Broth macrodilution test (BDT)

The experiments were based on the recommendations of the Manual of Clinical Microbiology associated with modifications published before [11, 13]. From each EO, 5% emulsions were made with either 0.2% of Polysorbate 80 or DMSO. After this, a serial twofold dilution was prepared from 50 to 0.0075 μL/mL. As control of the bacterial growth, neither an EO nor a detergent was added to the tubes. Test media containing 0.2% of Polysorbate 80 or DMSO were also used separately as emulsifier controls. DMSO was applied only in the case of *M. catarrhalis*, considering that this bacterium cannot tolerate Polysorbate 80. In the case of *Haemophilus* spp., we used *Haemophilus* test medium which consisted of 15 μg/mL hematin and NAD and 5% of yeast extract per mL. For the dilution series of antibiotics, a detergent was not used. 10 μL of an overnight bacterial culture (~ 4 × 10^7^ cells/mL) were added to each tube and incubated at 37 °C for 24 h. Then, in the case of *Streptococci* and *M. catarrhalis*, the tubes were plated out on 5% sheep blood agar and incubated again for 48 h. Chocolate agar was used for *Haemophilus* spp. The number of bacterial colonies was compared to the controls and then the values of the minimum bactericidal concentrations (MBC) and minimum inhibitory concentrations (MIC) were determined. The MBC is the lowest concentration of an antibacterial agent able to completely inhibit the growth of colonies. The MIC value is the concentration that could reduce the visible growth of bacteria in comparison with the controls. All tests were carried out in triplicate and under aerobic conditions.

### Vapor phase test (VPT)

The in vitro VPT were based on the method described by Kloucek et al. [14] with modifications of our previously published observations [11]. The test system was developed in a four-section Petri dish (PD, diameter 90 mm, VWR, Debrecen, Hungary) containing 5 ml of 5% sheep blood agar in the case of *Streptococci* and *M. catarrhalis*. *Haemophilus* spp. required chocolate agar with 15 μg/mL NAD supplementation. Test medium was not added into the upper lid of the PD. All bacteria were grown in solid test medium at 37 °C for 24 h before the assay and then inocula were made by dilution in sterile 0.9% saline to 10^5^ CFU/mL. Then three sections of the PD were inoculated with 20 μL of the selected bacterial suspensions. Different strains were spread into each section. The fourth compartment was left uninoculated as contamination control. Each EO sample was diluted with absolute ethanol (stock solutions: 0.5–195 μL/mL). 500 μL of stock solution was distributed on the surface of a sterile filter paper disc (thickness 0.18 mm, diameter 84 mm, Albet-Hahnemühle, Germany). The disc was placed on the separating wall of the PD after solvent evaporation. Therefore, there was approximately 2 mm distance between the disc and the inoculated agar surface. PDs were hermetically closed with Parafilm adhesive tape (Sigma Aldrich Ltd., Budapest, Hungary) to avoid the evaporation, and they were incubated at 37 °C for 48 h. After incubation, filter papers were removed and MIC values were determined. The MIC is the lowest concentration of an EO (expressed as μL of EO/free atmosphere above the growing microorganism) which can absolutely inhibit the visible growth of the bacteria. Filter paper discs containing absolute ethanol or left untreated were used as solvent and growth controls. All tests were carried out in triplicate.

## Results

### Headspace-solid phase microextraction - gas chromatographic mass spectrometry (HS-SPME – GC-MS) analysis

Chemical analyses of EOs were performed by GC-MS techniques. Identified compounds and percentage evaluation of the volatiles are shown in Table 1. In all samples, the amount of the detected components was above 93%. In accordance with the literature and our previous observations, the main volatiles of the headspace of cinnamon bark, eucalyptus, thyme, peppermint, and clove oil were *trans*-cinnamaldehyde (45.9%), 1,8-cineole (91.0%), thymol (46.1%), menthol (27.2%), and eugenol (66.9%), respectively. Beside the main constituents, γ-terpinene (3.2–6.5%), *p*-cymene (3.2–27.9%), menthone (19.8%), and β-caryophyllene (1.3–26.5%) were determined as minor components in the above mentioned EOs. In citronella oil, citronellal (42.3%), limonene (12.8%), and nerol (12.9%) were dominant. Scots pine oil contained *α*-pinene in higher amount (26.1%), but *β*-pinene (18.0%) and limonene (17.0%) were also detected in lower concentrations.

**Table 1:** Percentage composition of EOs by sHS-SPME-GC-MS analysis

Component	RI	Percentage of compounds (%)						
		1	2	3	4	5	6	7
*α*-Pinene	939	1.1	–	1.0	–	5.7	1.4	26.1
Camphene	951	–	–	2.2	–	–	–	7.9
*β*-Pinene	978	–	–	–	–	1.0	–	18.0
*β*-Myrcene	992	1.7	–	1.9	–	–	–	–
*α*-Phellandrene	1007	–	–	–	–	–	1.2	–
*α*-Terpinene	1017	–	–	1.9	–	–	–	–
*p*-Cymene	1026	–	–	27.9	–	6.1	–	3.2
*δ*-3-Carene	1031	–	–	–	–	–	–	14.4
Limonene	1044	–	12.8	–	–	8.2	–	17.0
1,8-Cineole	1046	17.4	–	3.7	–	11.1	91.0	–
*γ*-Terpinene	1060	–	–	6.5	–	–	4.4	3.2
Terpinolene	1093							3.3
Linalool	1104	–	1.0	3.5	–	6.7	–	–
Isopulegol	1150	1.1	1.0	–	–	–	–	–
Citronellal	1153	–	42.3	–	–	–	–	–
Menthone	1156	19.8	–	–	–	–	–	–
Isomenthone	1159	11.6	–	–	–	–	–	–
Anethole	1171	–	–	–	–	3.3	–	–
Menthol	1172	27.2	–	–	–	–	–	–
Isomenthol	1183	3.7	–	–	–	–	–	–
*α*-Terpineol	1190	–				2.2	–	1.3
Pulegone	1215	1.9	–	–	–	–	–	–
Citronellol	1226	–	8.9	–	–	–	–	–
Nerol	1230	–	12.9	–	–	–	–	–
*trans*-Cinnamaldehyde	1266	–	–	–	–	45.9	–	–
Bornyl acetate	1289	–	–	–	–	–	–	4.2
Thymol	1297	–	–	46.1	–	–	–	–
Isomenthyl acetate	1305	6.6	–	–	–	–	–	–
Citronellyl acetate	1353	–	4.6	–	–	–	–	–
Neryl acetate	1365	–	3.5	–	–	–	–	–
Eugenol	1373	–	–	–	66.9	1.4	–	–
*β*-Elemene	1394	–	3.0	–	–	–	–	–
*β*-Caryophyllene	1417	1.3	–	2.3	26.5	5.0	–	–
Cinnamyl acetate	1446	–	–	–	–	1.9	–	–
*α*-Humulene	1452	–	–	–	6.0	–	–	–
*β*-Cadinene	1473	–	2.6	–	–	–	–	–
*β*-Muurolene	1493	–	1.5	–	–	–	–	–
Total:		93.4	94.1	97.0	99.4	98.5	98.0	98.6

Table 1 shows the average content of volatile compounds which occurred in the EOs in more than 1% from 3 parallel measurements. The standard deviations (SD) were below 4.5%. 1. peppermint, 2. citronella, 3. thyme, 4. clove, 5. cinnamon bark, 6. eucalyptus, 7. scots pine. RI: Retention Index based on a homologous series of normal alkanes.

On the whole, we presume that the above mentioned components play the main role in the antibacterial activity of EOs in VP.

### Broth macrodilution test (BDT)

This method allowed us to detect the antibacterial activity of EOs in liquid media. MIC and MBC values of EOs were summarized in Tables 2 and 3. MIC values of general antibiotics are expressed in μg/mL in Table 4. Cinnamon bark, clove, citronella, and thyme presented the most potent inhibition against both Gram-negative and Gram-positive pathogens. The least sensitive strain to cinnamon and thyme was *S. pyogenes (*MIC: 0.41–0.43 mg/mL), while clove produced the lowest MIC value against this pathogen (MIC: 0.1 mg/mL). Among our tested materials thyme oil showed the most potent activity (MIC: 0.04 mg/mL) against *S. mutans*, which was followed by citronella, cinnamon bark, and clove. Citronella oil produced the lowest MIC value against *S. pneumoniae*. Peppermint presented inhibition against *Streptococcus* spp. in higher concentrations with MIC values in the range of 0.35–0.70 mg/mL.

**Table 2:** Antibacterial activity of EOs against *Streptococcus* spp. by broth macrodilution

Essential oil	*S. pyogenes*		*S. pneumoniae*		*S. mutans*	
	MIC	MBC	MIC	MBC	MIC	MBC
Cinnamon bark	0.41	0.81	0.06	0.13	0.20	0.41
Thyme	0.43	0.87	0.11	0.22	0.04	0.09
Clove	0.10	0.20	0.25	0.50	0.41	0.81
Peppermint	0.35	0.70	0.35	0.70	0.70	1.39
Citronella	0.17	0.34	0.09	0.17	0.17	0.34
Eucalyptus	2.82	5.64	1.41	2.81	0.70	1.41
Scots pine	1.35	2.71	0.68	1.35	1.35	2.71

*MIC* minimum inhibitory concentration, *MBC* minimum bactericidal concentration (in mg/mL)

**Table 3:** Antibacterial activity of EOs against *Haemophilus* spp. and *M. catarrhalis* by broth macrodilution

Essential oil	*H. influenzae*		*H. parainfluenzae*		*M. catarrhalis*	
	MIC	MBC	MIC	MBC	MIC	MBC
Cinnamon bark	0.06	0.13	0.06	0.13	0.10	0.20
Thyme	0.11	0.22	0.11	0.22	0.09	0.18
Clove	0.25	0.50	0.25	0.50	0.25	0.50
Peppermint	0.21	0.43	0.21	0.43	0.35	0.70
Citronella	0.21	0.42	0.11	0.21	0.11	0.21
Eucalyptus	1.41	2.81	0.70	1.41	2.81	5.64
Scots pine	1.35	2.70	0.34	0.68	0.34	0.68

*MIC* minimum inhibitory concentration, *MBC* minimum bactericidal concentration (in mg/mL)

**Table 4:** Antibacterial activity of antibiotics by broth macrodilution

Antibiotic	*S. pyogenes*	*S. pneumoniae*	*S. mutans*	*H. influenzae*	*H. parainfluenzae*	*M. catarrhalis*
	MIC_90_	MIC	MIC_90_	MIC	MIC	MIC_90_
Amoxicillin/clavulanic acid	–	–	0.8	–	–	0.2
Imipenem	0.25	0.8	3.1	–	–	0.2
Amikacin	–	–	–	3.1	1.6	–

MIC and MIC_90_: minimum inhibitory concentrations expressed in μg/mL

In the case of *Haemophilus* spp., cinnamon bark was the most effective (MIC: 0.06 mg/mL) oil, which was followed by thyme, peppermint, and clove. We observed that *H. influenzae* and *H. parainfluenzae* were similarly sensitive to these EOs, besides, *H. parainfluenzae* showed an increased sensitivity to citronella, scots pine, and eucalyptus oil. In lower amount (MIC: 0.34 mg/mL), scots pine also showed activity against *M. catarrhalis*. In general, it has been observed that eucalyptus showed activity only in higher concentrations (MIC: 0.7–2.82 mg/mL). Scots pine oil was active mostly in the case of our Gram-negative strains. In comparison with antibiotics, EOs produced inhibition only in higher concentrations. We must note that the effect of detergents did not influence our results.

### Antibacterial activity in vapor phase (VP)

Due to the absence of direct contact between the pathogen and EO, this method allows us to detect the antimicrobial potency of volatile components exclusively. As a result, MIC values were calculated and summarized in Table 5. They were determined considering the amount of EOs and the free airspace (L) in the Petri dish. As control, absolute ethanol did not show any antibacterial effect. Among the EOs, cinnamon bark was the most effective against all investigated pathogens with MIC values in the range of 15.62–90 μL/L. Above 90 μL/L, thyme oil effectively inhibited the bacterial growth of Gram-positive pathogens. Besides, thyme volatiles also showed potent inhibition against *Haemophilus* spp. and *M. catarrhalis*. In the case of peppermint and citronella oils, moderate activities were detected against Gram-negative strains (MIC: 31.25–75 μL/L), moreover, their effectiveness against *Streptococcus* species was also observed in higher amounts. In our test system, clove oil was active only above 90 μL/L. EO of scots pine did not show any inhibition in VP, except in the case of *H. influenzae*. Therefore, we presume that scots pine has bacteriostatic effect, and its MIC is probably higher than 1500 μL/L. In contrast, vapor of eucalyptus oil effectively inhibited the growth of *Haemophilus* spp. and *M. catarrhalis* in higher concentrations (MIC: 125–225 μL/L). In the case of *M. catarrhalis*, we found citronella and cinnamon bark oils equally active, which was followed by peppermint, thyme, and clove. Among our tested pathogens, *S. mutans* was the least sensitive to EO volatiles, in lower concentration only cinnamon bark performed potent inhibition (MIC: 90 μL/L) against this pathogen. In conclusion, we should highlight that Gram-negative strains were more sensitive to EO vapors: we detected higher MIC values against all Gram-positive bacteria.

**Table 5:** Antibacterial activity of cinnamon bark, thyme, clove, peppermint, citronella, eucalyptus, and scots pine oils by vapor phase test

Essential oil	*S. pyogenes*	*S. pneumoniae*	*S. mutans*	*H. influenzae*	*H. parainfluenzae*	*M. catarrhalis*
	MIC					
Cinnamon bark	75	75	90	15.62	15.62	25
Thyme	125	90	250	25	31.25	50
Clove	225	150	500	90	150	125
Peppermint	250	90	375	50	75	31.25
Citronella	125	50	250	50	62.5	25
Eucalyptus	> 1500	1200	> 1500	125	200	225
Scots pine	> 1500	> 1500	> 1500	500	> 1500	> 1500

*MIC* minimum inhibitory concentration [amount of EO in μL referred to airspace volume (L)]

## Discussion

Due to the hydrophobic character of EOs, classical microbiological tests are not relevant for detection of the antibacterial activity of these substances, thus, some modifications and development of new techniques is essential for this purpose. With BDT, we could detect the antibacterial effect of EOs in liquid medium; however, the inhibitory effect of volatiles could be determined with VPT [10]. The EOs application via inhalation is becoming more frequent nowadays, especially in the case of bacterial infections of the respiratory tract [7]. Classical antibacterial assays did not model the circumstances of inhalation; moreover, they usually focus on the activity of EOs via direct contact. In opposite, VPT detect the effect of gaseous phase produced by EO vapor and they can be easily combined with other techniques [8]. It should be highlighted that VPT can also be adapted to other different pathogens such as fungi and viruses [15–18]. According to the result of the microbiological assays, cinnamon bark, clove, thyme, peppermint, and citronella oils showed the most potent activity in both vapor and liquid systems. Therefore, they could be promising alternatives to support the current general treatment of bacterial infections. Their multicomponent composition gives them benefit in bacterial resistance; however, it simultaneously creates difficulties in their standardization and their effects’ proper comparison [19]. In liquid form, cinnamon bark was the most effective against *S. pneumoniae*, while thyme oil showed the best activity against *S. mutans*. In the case of *S. pyogenes*, clove oil produced the lowest MIC value, which was followed by citronella. Cinnamon bark and thyme were equally active against this pathogen, which was in accordance with previous results [5]. Interestingly, Mulyaningsih et al. reported inhibition of *S. pyogenes* by eucalyptus fruit oil in lower amount compared to our MIC values. Because we used EO distilled from the leaves, we suggest that the difference is probably caused by the compositions of the eucalyptus oils in the experiments [20]. Parallel to our results, a previous publication has reported the same inhibitory trend and cited strong antibacterial character of cinnamon bark and cinnamaldehyde against *S. mutans* [21]. Against this pathogen, antibiofilm activity of eucalyptus oil was also published [22]. Between *Haemophilus* spp., slight differences were observed between the EOs. Among our test materials, we detected the best inhibition in the case of cinnamon bark followed by thyme and clove, which was in accordance with previous observations [23, 24]. *H. parainfluenzae* was more susceptible to citronella, eucalyptus, and scots pine. However, eucalyptus oil and its vapor was previously reported as promising solutions against respiratory viruses (e.g. Influenza Virus type A and mumps virus), their antibacterial value in several studies were less potent than the antiviral effect [16, 25]. According to other reports, which support our findings, eucalyptus oil could be a more potent inhibitor of *Haemophilus* species in contrast with *S. pneumoniae* and *S. pyogenes* in liquid phase [5, 25]. *M. catarrhalis* was completely inhibited by thyme in low concentration; as well as cinnamon and citronella showed similar activity against this pathogen. The same potency was observed previously by Dorman et al. and Tanaka et al., in addition, EO components such as cinnamaldehyde, citronellal, thymol, eugenol, geraniol, limonene; *cis/trans* citral and *α*-terpineole also produced activity in their test systems [24, 26].

In VPT, Gram-negative pathogens were more sensitive to the EO treatment compared to Gram-positive bacteria. This observation was parallel to our experience in liquid media. Against all *Streptococcus* species, cinnamon bark vapor produced the lowest MIC value in the range of 75–90 μL/L. In the case of *S. pneumoniae*, the same inhibitory effect was observed than in BDT; however, we found scots pine vapor less active in gaseous phase. *S. pyogenes* was equally sensitive to citronella and thyme vapor, which was followed by clove and peppermint. Cinnamon bark volatiles were equally active against *H. influenzae* and *H. parainfluenzae*; however, slight differences were observed between these two bacteria considering the activity of thyme, citronella, and peppermint. Our observations were in accordance with previous reports [9]. Against the above mentioned Gram-negative bacteria, clove and eucalyptus oil performed inhibition only in relatively high concentrations (MIC: 90–200 μL/L). Houdkova et al. reported moderate activity of cinnamaldehyde and eugenol against *H. influenzae*; surprisingly, they did not report any differences between the activity of vapor and liquid form of these components [27].

*M. catarrhalis* was equally sensitive to the vapor of citronella and cinnamon bark; in contrast, clove and eucalyptus produced inhibition only in higher concentrations against this pathogen. Except in the case of *H. influenzae*, scots pine vapor did not manage proper inhibition below 1500 μL/L.

Table 6 summarizes the most potent EOs in liquid and vapor phase with MIC values below 0.5 mg/mL or 100 μL/L. In conclusion, we must highlight cinnamon bark as the most active EO in both in vitro systems. Besides, thyme, citronella, and peppermint oil and vapor also had strong antibacterial effect. At lower concentrations, clove oil was a more potent inhibitor in liquid phase; in vapor form it showed activity against *H. influenzae* only. Unfortunately, eucalyptus oil and its vapor were only active in higher concentrations.

**Table 6:** Comparison of antibacterial activity of EOs in liquid and vapor phase

Essential oil	Liquid phase	Vapor phase
Cinnamon bark	1, 2, 3, 4, 5, 6	1, 2, 3, 4, 5, 6
Thyme	1, 2, 3, 4, 5, 6	2, 4, 5, 6
Clove	1, 2, 3, 4, 5, 6	4
Peppermint	1, 2, 4, 5, 6	2, 4, 5, 6
Citronella	1, 2, 3, 4, 5, 6	2, 4, 5, 6
Eucalyptus	–	–
Scots Pine	5, 6	–

EOs were highlighted, if the MIC values were lower than 0.5 mg/mL or 100 μL/L. 1: *S. pyogenes* 2: *S. pneumoniae*, 3: *S. mutans*, 4: *H. influenzae*, 5: *H. parainfluenzae*, 6: *M. catarrhalis*

On the whole, we must emphasize that our EOs were more potent inhibitors in liquid form which is probably due to the direct contact with the pathogen. According to previous publications, EOs could interact with bacteria in many different ways such as alteration of the cell morphology, membrane permeability, and inhibition of enzymes [8, 19].

Several studies reported that Gram-positive bacteria were more sensitive to EOs and their components [28–30] than Gram-negative bacteria. Interestingly, we found that Gam-negative pathogens required less EO for their total inhibition in our both systems. We suggest that this is partly due to the fact that *S. mutans* forms biofilm, which enhance the resistance of this pathogen. Our observation was in correlation with the results of Inouye et al. [9]. The reason for this phenomenon is not fully understood; however, the authors pointed out that the outer membrane of *H. influenzae* may have an important function [9]. However, it should be taken into consideration that due to their lipophilic character they require effective formulation to achieve the proper activity in the respiratory tract. Thus, further development of effective and economical application of EOs’ special devices is indispensable in the future. [31–33].

## Conclusions

In the case of EOs, the in vitro antimicrobial assays should be optimized because of their hydrophobic character and multicomponent composition. Based on our results, we suggest that VPT provides the best detection for the activity of EOs based on gaseous contact. However, BDT is one of the most suitable direct-contact assays. On one hand, only the optimized BDT and VPT are able to provide trustworthy results about the antimicrobial effect of EOs. On the other hand, the evaluation of antibacterial activity it should be taken into consideration that EOs have different characters in liquid form or in VP which results in diverse biological activity. We conclude that cinnamon bark oil possess the strongest antibacterial activity against all the respiratory tract pathogens used in our study. On the whole, it should be highlighted that cinnamon, thyme, peppermint, and citronella also showed potent antimicrobial activity in vapor and in liquid form; in contrast, clove oil was more potent inhibitor in liquid phase. Finally, in vitro and clinical studies are also required to calculate the effective doses of EOs, determine the interactions between the components and reveal their toxicity.
